# Neuronal and glial cell biology

**DOI:** 10.1016/j.conb.2009.10.013

**Published:** 2009-10

**Authors:** Peter Brophy, Kang Shen

**Affiliations:** Centre for Neuroregeneration, University of Edinburgh, Chancellor's Building, 49 Little France Crescent, Edinburgh EH16 4SB, UK; Department of Biological Sciences, Stanford University, 371 Serra Mall, Herrin Labs Room 144, Stanford, CA 94305-5020, USA

## Introduction

The wiring of the nervous system is arguably the most challenging question in developmental biology. In recent years there has been tremendous progress in elucidating how the axons of neurons seek out, recognize, and establish connections with their targets in the developing nervous system. This process can be broken down into cell fate determination, axon/dendrite guidance, synapse formation, and activity dependent modification of synaptic circuit.

Once axons have reached their targets, it is now understood that a key aspect of developmental plasticity is the refining and sculpting of connections between neurons in order to enhance the function of neuronal networks. A later event in the development of the vertebrate nervous system is the optimization of these networks by accelerating the speed of nerve impulse conduction. This is the role of the glia, particularly the myelin-forming glia-oligodendrocytes in the CNS and Schwann cells in the PNS. They achieve this by inducing the assembly of macromolecular complexes at the nodes of Ranvier, which are highly enriched in voltage-gated sodium channels. A second key role for glia is in supporting the health and survival of axons. This aspect of glial function has become particularly evident from studies on demyelinating diseases in which loss of axonal contact is associated with axonal degeneration.

## MicroRNAs in brain development and physiology

One of the most exciting advances in our understanding in gene regulation and translational control is the discovery of microRNAs. We have just started to collect evidence that microRNAs are involved in the development and physiology of the nervous system. Coolen and Bally-Cuif review the recent literature on the roles of microRNAs in early developmental processes including cell fate determination, neurogenesis, and patterning, as well as microRNAs’ involvement in the function of mature CNS and under disease conditions.

## Function of axon guidance molecules in synapse formation

Over the last 20 years, our understanding of axon guidance was revolutionized by the discovery of multiple families of guidance molecules and receptors. Interestingly, many of the molecules that were discovered for their roles in axon guidance are now also implicated in the process of synapse formation. This might not seem to be a surprise because axon guidance and synapse formation occur in similar developmental stages. However, the differences in the cell biological events in these two processes demand more answers on how similar receptor–ligand interactions can trigger vastly different intracellular events in axon guidance and synapse formation. Chen and Cheng, and Eroglu summarize our current understandings on this topic.

## How oligodendrocytes differentiate

We now have a fairly clear picture of the embryological origin of both oligodendrocytes and Schwann cells. Hence, there is an increased emphasis on understanding the genetic specification of these cells, not least because the adult CNS contains a large pool of apparently dormant oligodendrocyte progenitors, which could, in principle, be recruited to repair demyelinated lesions. Inherent genomic variation may of course contribute to risk in diseases such as MS. However, it is becoming equally clear from genome wide association studies that simply focusing on mutations/polymorphisms alone is unlikely to provide the key insights since studies conducted thus far seem to indicate that they account for no more than around 5% of risk in several common diseases. In this context, it may be that epigenetic factors have a key influence. Therefore, it is of great interest that Li, He, Richardson, and Casaccia find that both transcriptional and epigenetic regulatory factors play a vital role in oligodendrocyte differentiation. Techniques for analyzing the mammalian epigenome, although still at an early stage of development, are attracting considerable attention and it is likely that the interaction between these levels of regulation, the ‘two-pronged’ approach as described by these authors, will help us to understand not only how oligodendrocytes differentiate, but also why repair is so inefficient in the adult human CNS.

## Mechanisms of myelination

The work of Colman *et al.* on myelin biosynthesis by oligodendrocytes (Ref. [37] in Monk and Talbot) was the first demonstration in mammalian cells of the translocation and local translation of mRNA. There has been considerable debate about the function of this phenomenon in myelination so the demonstration that the kinesin motor Kif1b is a key regulator of this translocation in a zebrafish screen (Lyons *et al.*, Ref. [32] in Monk and Talbot) is highly significant, not least because recent genetic studies have implicated the gene that encodes Kif1b in susceptibility to multiple sclerosis (Aulchenko *et al.*, Ref. [41] in Monk and Talbot). This work also provides important evidence as to why translocation of the mRNA that encodes myelin basic protein is important in the assembly of the myelin sheath since premature synthesis seems to provoke aberrant, ectopic compaction of the myelin membrane.

## Roles of glia in the development and function of synapses

Recent studies uncovered new roles for glia in the development and function of synapses (Bolton and Eroglu). Contact-mediated signals from glia instruct dendrites to become receptive to synaptic partners. Glia-derived factors coordinate the assembly presynaptic structures and the precise apposition of presynaptic and postsynaptic specializations. Glial cells stimulate the process of synapse formation *in vitro* and *in vivo*. Glial cells also provide cues that are required for synaptic maturation and remodeling of spines both during development and in the adult.

## Axoglial interaction and axonal degeneration

Although it is uncontroversial that loss of glial contact promotes axonal degeneration, it was a great surprise that even apparently modest alterations in oligodendrocyte cell biology might affect axonal function (Edgar and Nave). The health of other glial cells such as astrocytes and microglia is also clearly implicated in the survival of neurons in other neurodegenerative diseases such as ALS. One model proposed by these authors is that the energy metabolism of axons is affected by glial contact, presumably mediated via mitochondria. This suggests that we need to learn much more about the balance between the electrophysiological necessity of sequestering the axon from its immediate environment through myelination and its essential requirement for nutrients from the same environment.

## Mechanisms of glial and axonal protection

Neurology has been a strong competitor with economics for the title ‘the dismal science’. Indeed it is often referred to as a diagnostic subspecialty, possibly due to the relatively small number of truly efficacious therapies available, at least compared to some other branches of medicine. Clearly, in order to turn this state of affairs around, a mechanistic understanding of neurological disease is required. A major feature of degenerative neurological disease, both in the CNS and PNS, is the production of mutant proteins whose folding and associations are believed to disrupt normal neuronal and glial function. Recent developments in our understanding of how misfolded proteins are handled by cells, including the unfolded protein response, have had immediate and obvious relevance to potential therapeutic interventions in both the CNS and PNS. Gow and Wrabetz show how studying these fundamental cell biological processes in mouse models of disease is likely to reveal targets for therapeutic intervention.

## Conclusions

While we are likely only seeing the tip of the iceberg, it is evident that the crosstalk between neurons and glia cells happens at many levels both in the developing as well as in the mature nervous system. Neuronal complexity in the mature nervous system is taken for granted but it is increasingly recognized that glia also have diverse functions. Indeed NG2 positive glia are exceptionally abundant but their role is still far from clear. Understanding the interactions between these various cell types will be the key to uncover the mysteries of the brain.

